# Polycystic ovary syndrome and miscarriage risk: dual evidence from cohort study and Mendelian randomization

**DOI:** 10.3389/fendo.2026.1854797

**Published:** 2026-07-24

**Authors:** Huihuang Chi, Zhe Yang, Jing Dong, Jiexue Pan, Jianzhong Sheng, Hefeng Huang, Zuwei Yang

**Affiliations:** 1 Institute of Reproduction and Development, Shanghai Key Laboratory of Reproduction and Development, Obstetrics and Gynecology Hospital, Fudan University, Shanghai, China; 2 Shanghai Key Laboratory of Female Reproductive Endocrine Related Diseases, Shanghai, China; 3 Medical Center of Diagnosis and Treatment for Cervical Disease, Obstetrics and Gynecology Hospital, Fudan University, Shanghai, China; 4 Institute of Medical Genetics, Key Laboratory of Reproductive Genetics (Ministry of Education), and Women’s Hospital, Zhejiang University School of Medicine, Hangzhou, Zhejiang, China

**Keywords:** assisted reproductive technology (ART), Mendelian randomization, miscarriage, polycystic ovary syndrome, pregnancy outcomes

## Abstract

**Objective:**

To investigate the association between polycystic ovary syndrome (PCOS) and miscarriage risk and to explore the potential causal effect.

**Methods:**

We conducted a retrospective cohort study of 445 women who conceived via *in vitro* fertilization/intracytoplasmic sperm injection, comprising 191 PCOS women and 254 controls. The primary outcome was miscarriage. Logistic regression adjusted for maternal age, body mass index, endometrial thickness on hCG day, the number of embryos transferred, embryo transfer/fertilization methods yielded adjusted odds ratios (aORs) with 95% confidence intervals (CIs). Subgroup analyses assessed the association of PCOS with miscarriage across key demographic and clinical variables. Additionally, two-sample Mendelian randomization (MR) analyses were performed to evaluate the causal effect of PCOS on miscarriage risk.

**Results:**

Compared with controls, women with PCOS had higher risks of miscarriage (18.4% vs. 11.0%, p = 0.029) and biochemical pregnancy rates (5.2% vs. 1.2%, p = 0.012), alongside lower live birth rates (73.3% vs. 81.9%, p = 0.03). The increased risk was mainly driven by early miscarriage (14.2% vs. 8.3%, p = 0.048). In logistic regression, PCOS was significantly associated with miscarriage in univariate analysis (OR = 1.795, 95% CI: 1.049–3.072, p = 0.033), and remained an independent risk factor after multivariable adjustment (aOR = 1.851, 95% CI: 1.019–3.362, p = 0.043). Subgroup analyses revealed consistent trends, though some strata lacked statistical significance. The primary MR analysis using the inverse-variance weighted (IVW) method demonstrated a significant causal effect of PCOS on the risk of sporadic miscarriage (OR = 1.08, 95% CI: 1.01–1.14, p = 0.0203). The replicated MR analysis yielded similar effect sizes, and genetically predicted PCOS liability aligned with the cohort study results. Sensitivity analyses showed no significant heterogeneity or horizontal pleiotropy, supporting the robustness of the results.

**Conclusion:**

Integrating dual evidence from clinical cohort and MR analyses, PCOS is associated with increased miscarriage risk, potentially independent of traditional risk factors. These findings underscore the importance of early pregnancy management in women with PCOS and establish a basis for future research into underlying mechanisms and potential interventions.

## 1 Introduction

Polycystic ovary syndrome (PCOS) is one of the most prevalent endocrine-metabolic disorders in women of reproductive age, with an estimated global prevalence of 6–10% and recognition as a leading cause of female infertility (1). Characterized by menstrual irregularities, hyperandrogenism, ovulatory dysfunction, and metabolic disturbances (e.g., insulin resistance, obesity), PCOS profoundly influences pregnancy outcomes (2). Women with PCOS face elevated risk of multiple adverse gestational events, including gestational diabetes mellitus, gestational hypertension, preeclampsia, preterm birth, and miscarriage (3). Among these, miscarriage is particularly common, imposing significant physical and psychological burdens, heightened healthcare utilization, and potential compromising future fertility. Thus, miscarriage represents a critical reproductive outcome requiring focused attention in both clinical practice and research regarding PCOS (4).

Despite extensive research, the relationship between PCOS and miscarriage remains controversial. Observational studies and meta-analyses have consistently reported an elevated incidence of pregnancy loss in women with PCOS (5, 6). A recent large-scale meta-analysis reported a 49% higher miscarriage risk in PCOS women compared with non-PCOS controls, a finding that persists across both natural and assisted reproductive technology-conceived pregnancies and remains significant even after adjustment for age and elevated body mass index (BMI) in rigorously matched studies (7). Nevertheless, some studies have reported inconsistent findings. The 2012 Amsterdam ESHRE/ASRM consensus explicitly stated that miscarriage rates are not increased in women with PCOS who conceive naturally, and that miscarriage rates following ovulation induction are comparable to those observed in other infertile populations (8). Furthermore, one study has found that PCOS women undergoing *in vitro* fertilization (IVF) had a significantly higher rate of biochemical pregnancy loss, whereas the clinical miscarriage rate was not significantly different compared with non-PCOS patients (9). In addition, inherent limitations of observational designs, such as confounding bias and reverse causality (10), preclude definitive causal conclusions. Furthermore, metabolic comorbidities frequently co-occurring with PCOS, including obesity and insulin resistance, may further exacerbate this risk (11). Notably, some studies suggest that after adjusting for confounders such as body weight and treatment modality, the independent association between PCOS and spontaneous miscarriage is attenuated (12), further complicating the interpretation of this association. Consequently, a critical gap remains: does PCOS independently drive miscarriage risk, or is the observed association primarily mediated by overlapping metabolic and endocrine disturbances? Resolving this uncertainty is essential to clarify the clinical relevance of PCOS in pregnancy loss.

In this context, Mendelian randomization (MR) offers a valuable methodological approach for causal inference. By leveraging genetic variants as instrumental variables, MR minimizes confounding and reverse causality, offering robust evidence of causal relationships between exposures and outcomes (13). Yet, MR studies specifically investigating the causal effect of PCOS on miscarriage remain scarce, and the underlying causal mechanisms are still poorly understood.

To address these gaps, we combined two complementary methodological approaches: a retrospective clinical cohort study to assess the observational association, and two-sample MR based on large-scale genome-wide association studies (GWAS) to infer the potential causal relationship between PCOS and miscarriage risk. This study aims to evaluate the association between PCOS and miscarriage risk, and to explore its potential causal basis. By synthesizing evidence from both conventional clinical epidemiology and genetic epidemiology, we seek to clarify the etiological underpinnings of miscarriage in PCOS, inform risk stratification, and guide clinical management for this high-risk population.

## 2 Materials and methods

### 2.1 Study design and participants

This study was a single-center retrospective cohort analysis conducted at the Reproductive Medicine Center of Women’s Hospital, Zhejiang University School of Medicine, from January 1, 2020, to December 31, 2024, and was approved by the institution’s ethics Review Board. Clinical data of 1,090 women who underwent IVF/intracytoplasmic sperm injection (ICSI) cycles were retrospectively reviewed. Eligible participants were women who achieved their first pregnancy following IVF/ICSI embryo transfer at our center. The inclusion criteria for this study were as follows: (1) age between 20 and 40 years; (2) undergoing IVF/ICSI treatment; (3) retrieval of ≥3 oocytes and transfer of 1–2 embryos; (4) availability of complete pregnancy records. The exclusion criteria included: (1) ovarian cysts or tumors, ovarian damage caused by surgery or torsion, or premature ovarian insufficiency; (2) endometriosis, uterine malformations (including unicornuate uterus, septate uterus, didelphys uterus, or bicornuate uterus), adenomyosis, submucosal fibroids, or intrauterine adhesions; (3) hydrosalpinx, pelvic inflammatory disease, autoimmune disease, chronic inflammatory disorders, or other clinically apparent inflammatory conditions; (4) a history of recurrent pregnancy loss; and (5) receipt of preimplantation genetic testing (PGT).

**Figure 1:**
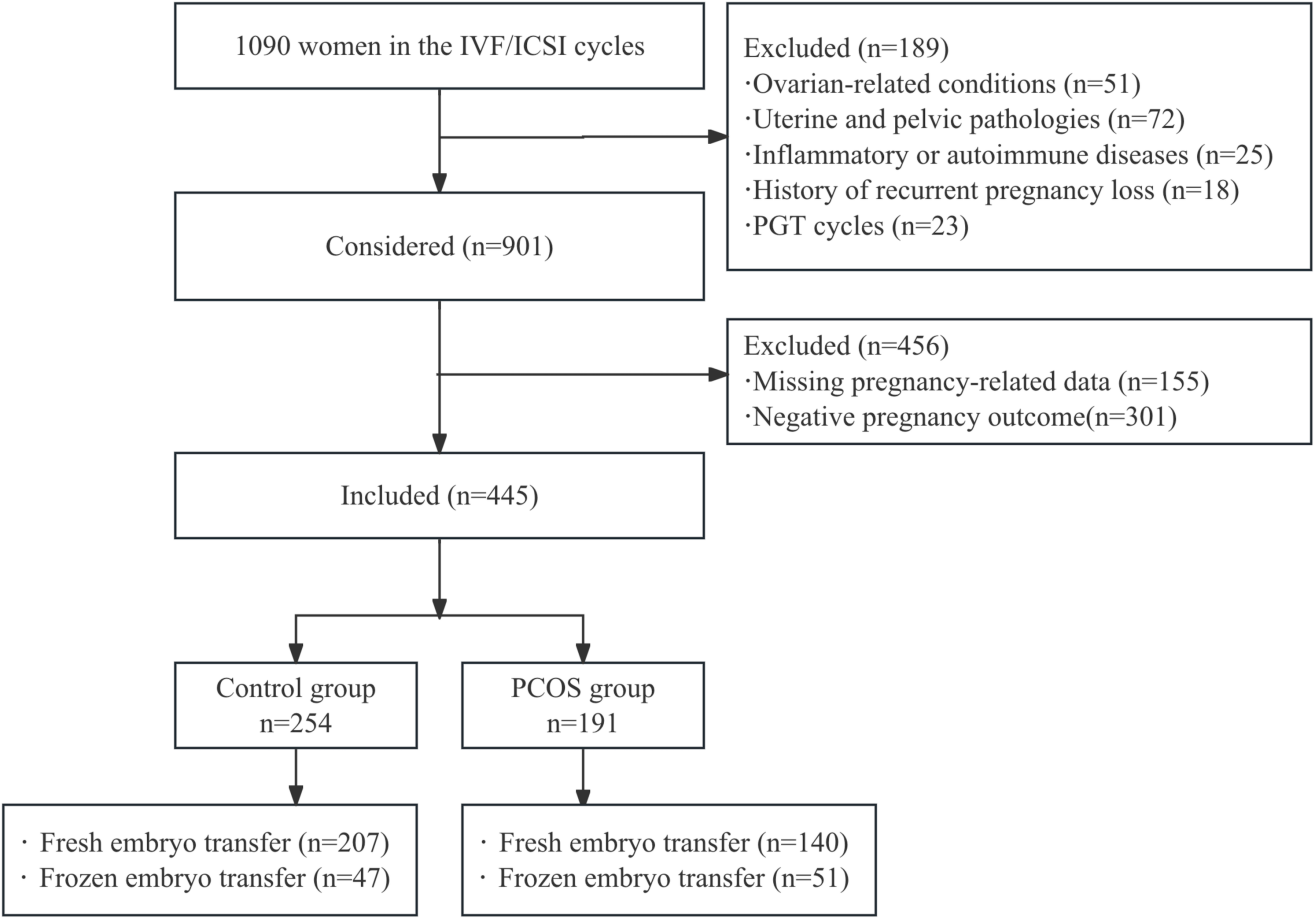
Flowchart of patient selection in the control and PCOS groups.

PCOS was diagnosed according to the Rotterdam criteria (14), which require the presence of at least two of the following three features after excluding other potential causes such as hyperprolactinemia, androgen-secreting tumors, Cushing’s syndrome, and congenital adrenal hyperplasia: (1) oligo-ovulation or anovulation; (2) clinical or biochemical hyperandrogenism; (3) polycystic ovarian morphology on transvaginal ultrasound. Oligomenorrhea was defined as fewer than eight menstrual cycles per year or a menstrual interval longer than 35 days, while amenorrhea was defined as the absence of menstruation for at least six consecutive months. Clinical hyperandrogenism was defined as a modified Ferriman–Gallwey score of ≥4 to ≥8, and biochemical hyperandrogenism was defined as elevated total or free testosterone, or calculated indices of free testosterone. Polycystic ovarian morphology was defined as the presence of ≥12 follicles per ovary and/or ovarian volume ≥10 cm^3^. Women undergoing IVF/ICSI due to tubal factor infertility were initially enrolled as the control group. All control participants had regular menstrual cycles, normal androgen levels, and no evidence of polycystic ovarian morphology on ultrasound examination. According to the above inclusion and exclusion criteria, a total of 254 women with tubal factor infertility (other infertility factors were excluded) and 191 women with PCOS were ultimately included in the final analysis (**Figure 1**).

### 2.2 IVF and embryo transfer protocols

All patients’ information, including baseline characteristics, cycle parameters, and pregnancy outcomes, were extracted from the electronic medical records. Controlled ovarian stimulation was performed according to standard clinical practice. Briefly, individualized stimulation protocols, including gonadotropin-releasing hormone agonist or antagonist regimens, were selected based on patients’ age, ovarian reserve, and endocrine profiles. Follicular development was monitored by serial transvaginal ultrasound and serum hormone measurements. When at least two dominant follicles reached ≥18 mm in diameter, final oocyte maturation was triggered with human chorionic gonadotropin (hCG), and oocyte retrieval was scheduled 36 hours thereafter under ultrasound guidance. Retrieved oocytes were inseminated by conventional IVF or ICSI according to sperm quality. Embryos were cultured in pre-equilibrated culture media under controlled laboratory conditions, and embryologists assessed embryo development on days 3 and 5/6 post-insemination. Embryo quality was evaluated morphologically according to established (15), high-quality embryos were defined based on developmental stage–specific morphological parameters. Cleavage-stage embryos were evaluated according to cell number, fragmentation, and blastomere symmetry, whereas blastocysts were graded using the Gardner scoring system, which assesses expansion degree, inner cell mass, and trophectoderm. Blastocysts graded ≥3BB were considered high quality. Based on these evaluations, high-quality cleavage-stage embryos or blastocysts were selected for transfer.

Based on ovarian response and endometrial conditions, either a fresh embryo transfer (ET) or a frozen-thawed embryo transfer (FET) was performed. One or two embryos were transferred depending on patient age and embryo quality. In fresh ET cycles, luteal support with vaginal progesterone was initiated on the day of oocyte retrieval and continued until pregnancy confirmation. In FET cycles, endometrial preparation was performed prior to transfer, with transvaginal ultrasound used to assess endometrial thickness and morphology. Embryo transfer was conducted when endometrial thickness reached ≥8 mm with a trilaminar pattern. Luteal support in FET cycles was administered according to the specific endometrial preparation protocol, with progesterone supplementation tailored to the regimen used (e.g., natural or artificial cycles), and was continued until fetal cardiac activity was confirmed.

### 2.3 Pregnancy outcomes

The primary outcome of the study was miscarriage, which included early miscarriage (pregnancy loss before 12 weeks of gestation) and late miscarriage (pregnancy loss between 12 and <28 weeks of gestation). Biochemical pregnancy, live birth, and ectopic pregnancy were assessed as secondary outcomes Biochemical pregnancy was defined as a positive serum or urinary hCG test without ultrasound evidence of intrauterine pregnancy during follow-up. Live birth was defined as the delivery of a fetus at ≥28 weeks of gestation with signs of life after birth. Ectopic pregnancy was defined as a pregnancy in which implantation occurred outside the uterine cavity.

### 2.4 Statistical analyses

Descriptive statistics were used to summarize maternal and cycle characteristics. Normally distributed continuous variables were presented as mean ± standard deviation, non-normally distributed variables as median with interquartile range, and categorical variables as frequencies and percentages. Between-group differences were assessed using independent-samples t-tests, nonparametric tests, or chi-square tests, as appropriate.

Univariable logistic regression was performed to estimate odds ratios (ORs) and 95% confidence intervals (CIs) of pregnancy outcomes associated with PCOS. Multivariable logistic regression analysis was conducted to adjust for potential confounders, including maternal age, BMI, endometrial thickness on the day of hCG trigger, number of embryos transferred, embryo transfer method, and fertilization method. To further explore the associations in different clinical subgroups, analyses were stratified by some covariates. Given the exploratory nature of these analyses, no correction for multiple testing (e.g., Bonferroni adjustment) was applied, and the reported *p*-values are nominal. All tests were two-sided, with p<0.05 considered statistically significant. Statistical analyses were performed using SPSS (version 27.0 for Mac OS).

### 2.5 Mendelian randomization analyses

#### 2.5.1 Data source

Two-sample MR is a causal inference approach that uses genetic variants associated with an exposure as instrumental variables to estimate the causal effect of the exposure on an outcome. This method exploits summary statistics from large-scale GWAS of exposures and outcomes, thereby reducing biases commonly encountered in observational studies.

To enhance the robustness and reproducibility of causal inference, we conducted two-sample MR analyses using a primary dataset and subsequently validated the findings through a replication analysis with an independent GWAS dataset (**Supplementary Table 1**). To mitigate bias from population stratification, both analyses were restricted to individuals of European ancestry.

In the primary MR analysis, genetic instruments for PCOS were obtained from a genome-wide association meta-analysis including 10,074 PCOS cases and 103,164 controls from 7 cohorts of European descent (16). Summary statistics for the outcome, spontaneous abortion, were sourced from the FinnGen R11 dataset (finngen_R11_O15_ABORT_SPONTAN), comprising 20,775 spontaneous abortion cases and 180,063 controls of European ancestry. We assessed potential sample overlap between these datasets. Six of the seven PCOS cohorts were recruited from non-Finnish European countries or the United States, with no expected overlap with the FinnGen cohort. For the 23andMe cohort, formal overlap quantification via MRlap was not feasible due to restricted individual-level data access, but overlap is expected to be minimal given its predominantly US participant pool. A Type I error simulation (rate = 0.05, under a conservative 25% assumed overlap) confirmed no inflated false-positive rate or meaningful bias in our causal estimates.

To further validate the robustness of our findings, a replication MR analysis was performed using an independent data source, in which PCOS-associated genetic variants were obtained from the FinnGen R11 dataset (finngen_R11_E4_PCOS), including 1,909 cases and 241,998 controls. Summary statistics for sporadic miscarriage were extracted from a previously published GWAS comprising 49,996 cases and 174,109 controls (GWAS ID: GCST011888), as originally reported by Laisk et al. (17). We verified against the original publication that this miscarriage GWAS included no Finnish participants, so there is no risk of sample overlap in the replication stage.

#### 2.5.2 Selection of genetic instruments

In the primary MR analysis, single-nucleotide polymorphisms (SNPs) associated with PCOS were selected at a genome-wide significance threshold (*p* < 5×10^-8^).To obtain sufficient instrumental variables(IVs), in the secondary MR analysis, however, only six SNPs met this threshold, which fell short of the minimum requirement of at least 10 eligible instrumental variables for a valid MR analysis. Therefore, a more lenient threshold (*p* < 5×10^-6^) was applied, yielding 10 SNPs for subsequent analysis (18, 19). The selected SNPs we correlated strongly with the exposure factors and were assessed for their association with the outcome events. To ensure independence, all SNPs were clumped to avoid the linkage disequilibrium under a strict clump window (r^2^<0.001 and kb<10,000). F statistics were calculated to estimate the sample overlap effect and weak instrument bias considering the relatively relaxed threshold, and an F<10 was considered dubious bias (20). Only SNPs with F statistics > 10 were retained. Palindromic SNPs with ambiguous strand orientation were excluded from the analyses. Details of the finally identified IVs are presented in **Supplementary Tables 2**, **3**.

#### 2.5.3 MR analyses

We employed several conventional MR methods, including inverse variance weighting (IVW), weighted median (WM), MR−Egger, simple mode, and weighted model (21). As the primary analytical method, IVW combines the causal estimates of multiple SNPs through meta-analysis to obtain an overall effect, assuming all SNPs are valid instrumental variables and that no horizontal pleiotropy is present. The WM method sorts the causal estimates from all SNPs and takes the weighted median, delivering robust results when at least 50% of the weight comes from valid instruments. MR−Egger estimates the causal effect via its regression slope, while its intercept provides an assessment of directional horizontal pleiotropy. Consistent with standard MR practice, in this study, we adopted IVW as the primary method for causal estimation, with other MR methods serving as complementary methods.

Evaluating heterogeneity and horizontal pleiotropy represents a critical step in MR analysis. In this study, heterogeneity testing was conducted to determine whether to apply a fixed-effects or random-effects model. The absence of heterogeneity supports the reliability of the results. Horizontal pleiotropy refers to the possibility that instrumental variables affect the outcome through pathways other than the exposure of interest. If not properly addressed, horizontal pleiotropy may violate the core assumptions of MR and introduce bias into the causal inference. Heterogeneity was assessed using Cochran’s Q statistic, with a p-value < 0.05 indicating significant heterogeneity. Horizontal pleiotropy was evaluated by examining the intercept of the MR-Egger regression; a non-zero intercept with a p-value < 0.05 was considered evidence of directional horizontal pleiotropy. Additionally, a leave-one-out sensitivity analysis was performed to examine whether the overall results were driven by any SNP, thereby ensuring that the conclusions were not unduly influenced by individual genetic variants.

The two-sample MR analyses were performed using R software (version 4.5.0) along with the relevant MR packages (TwoSampleMR and Mendelian Randomization). Causal estimates were given as ORs with 95% CIs, and *p* < 0.05 was considered statistically significant.

## 3 Results

### 3.1 Baseline characteristics

A total of 445 women undergoing IVF/ICSI-conceived pregnancies were enrolled in this retrospective cohort, comprising 191 women diagnosed with PCOS and 254 controls (**Figure 1**). Baseline characteristics of both groups are summarized in **Table 1**. No significant differences were observed in maternal age (29.40 ± 3.40 vs. 29.26 ± 2.89 years, *p* = 0.640) or the number of embryos transferred (1.90 ± 0.30 vs. 1.89 ± 0.31, *p* = 0.797) between groups. However, women with PCOS had a significantly higher BMI compared to the control group (23.65 ± 3.85 vs. 21.57 ± 2.63 kg/m², *p* < 0.001) and a longer duration of infertility (4.44 ± 2.61 vs. 3.77 ± 2.64 years, *p* = 0.008). PCOS patients displayed characteristic reproductive endocrine abnormalities, including significantly longer menstrual cycles (100.54 ± 88.27 vs. 29.72 ± 2.83 days, *p* < 0.001), an increased luteinizing hormone (LH)/follicle-stimulating hormone (FSH) ratio (1.66 ± 1.03 vs. 0.72 ± 0.28, *p* < 0.001), and higher testosterone levels (1.54 ± 0.76 nmol/L vs. 0.89 ± 0.43 nmol/L, *p* < 0.001). Furthermore, the PCOS group had a significantly higher antral follicle count (AFC; 21.01 ± 4.46 vs. 11.02 ± 3.36, *p* = 0.001) but a thinner endometrial thickness on the day of hCG administration (10.41 ± 2.41 vs. 10.91 ± 2.40 mm, *p* = 0.030) than controls.

**Table 1:** Clinical characteristics of women in the control and PCOS groups.

Maternal characteristics	Control (n = 254)	PCOS (n = 191)	*P*-value
Maternal age (year)	29.40 ± 3.40	29.26 ± 2.89	0.640
Maternal BMI (kg/m^2^)	21.57 ± 2.63	23.65 ± 3.85	<0.001
<18.5	24 (9.6%)	8 (4.2%)	
18.5-24.9	190 (76.0%)	116 (61.4%)	
25-29.9	35 (14.0%)	50 (26.5%)	
>=30	1 (0.4%)	15 (7.9%)	
Cycle length (day)	29.72 ± 2.83	100.54 ± 88.27	<0.001
Infertility duration, years	3.77 ± 2.64	4.44 ± 2.61	0.008
Day-3 LH (IU/L)	4.62 ± 1.69	9.60 ± 6.27	<0.001
Day-3 FSH (IU/L)	6.62 ± 1.57	5.79 ± 1.40	<0.001
Day-3 LH/FSH	0.72 ± 0.28	1.66 ± 1.03	<0.001
Day-3 E2 (pmol/L)	133.08 ± 52.18	141.16 ± 72.99	0.203
PRL (ng/ml)	17.18 ± 9.60	16.17 ± 11.23	0.319
Day-3 TT (nmol/L)	0.89 ± 0.43	1.54 ± 0.76	<0.001
Day-3 DHEA (µmol/L)	6.87 ± 2.29	7.76 ± 3.01	0.011
AFC	11.02 ± 3.36	21.01 ± 4.46	0.001
Serum E2 level (pmol/ml) on hCG day	14083.51 ± 8061.62	16912.77 ± 10246.21	0.002
Endometrial thickness (mm) on hCG day	10.91 ± 2.40	10.41 ± 2.41	0.030
Number of follicles >14mm	9.66 ± 3.88	14.19 ± 5.90	<0.001
No. of oocyte retrieved	13.64 ± 5.98	17.79 ± 8.36	<0.001
No. of fertilized occytes	10.98 ± 5.47	13.13 ± 7.39	<0.001
No. of cleavage embryos	9.39 ± 4.88	11.94 ± 6.84	<0.001
No. of high quality embryos	3.34 ± 3.26	5.25 ± 5.78	<0.001
No. of embryos transferred	1.9 ± 0.30	1.89 ± 0.31	0.797
Miscarriage rate (%)	11.0 (28/254)	18.4 (35/191)	0.029
Early miscarriage rate (%)	8.3 (21/254)	14.2 (27/191)	0.048
Late miscarriage rate (%)	2.8 (7/254)	4.2 (8/191)	0.407
Live birth rate (%)	81.9 (208/254)	73.3 (140/191)	0.03
Ectopic abortion rate (%)	5.9 (15/254)	3.1 (6/191)	0.173
Chemical pregnancy rate (%)	1.2 (3/254)	5.2 (10/191)	0.012

BMI, body mass index; Day 3, the third day of spontaneous menstrual cycle; LH, luteinizing hormone; FSH, follicle stimulating hormone; E2, estradiol; PRL, prolactin; TT, total testosterone; DHEA, Dehydroepiandrosterone; AFC, antral follicle count; hCG, human chorionic gonadotropin.

In terms of pregnancy outcomes, women with PCOS had a significantly higher overall miscarriage rate than controls (18.4% vs. 11.0%, *p* = 0.029). This difference was primarily driven by an increased early miscarriage rate (14.2% vs. 8.3%, *p* = 0.048), whereas the rate of late miscarriage did not differ significantly between the two groups *(p* = 0.407). Correspondingly, the live birth rate was lower in the PCOS group (73.3% vs. 81.9%, *p* = 0.030). Ectopic pregnancy rates did not differ significantly (*p* = 0.173). Notably, the chemical pregnancy rate was also higher in PCOS patients (5.2% vs. 1.2%, *p* = 0.012).

### 3.2 Pregnancy outcomes stratified by embryo transfer method

Pregnancy outcomes were further compared between the two groups according to embryo transfer methods (**Table 2**). Among all cycles, 347 (78.0%) underwent ET, while 98 (22.0%) underwent FET. No statistically significant differences were observed in miscarriage, live birth, or ectopic pregnancy rates (all *p* > 0.05). For fresh ET, the PCOS group exhibited trends toward higher total miscarriage (18.6% vs. 12.0%, *p* = 0.094) and early miscarriage rates (15.0% vs. 9.2%, *p* = 0.096), along with a lower live birth rate (72.1% vs. 80.7%, *p* = 0.063). Similar directional trends were noted in FET. A notable exception was the chemical pregnancy rate, which was significantly elevated in the PCOS group undergoing fresh ET compared to controls (5.7% vs. 1.0%, *p* = 0.009).

**Table 2:** Pregnancy outcomes of women in the control and PCOS groups according to different embryo transfer methods.

Pregnancy outcomes	Fresh embryo transfer (n=347)			Frozen embryo transfer (n=98)		
	Control (n=207)	PCOS (n=140)	*P*-value	Control (n=47)	PCOS (n=51)	*P*-value
Miscarriage rate (%)	12.0 (25/207)	18.6 (26/140)	0.094	6.4 (3/47)	17.6 (9/51)	0.089
Early miscarriage rate (%)	9.2 (19/207)	15.0 (21/140)	0.096	4.2 (2/47)	11.8 (6/51)	0.175
Late miscarriage rate (%)	2.9 (6/207)	3.6 (5/140)	0.726	2.1 (1/47)	5.9 (3/51)	0.348
Live birth rate (%)	80.7 (167/207)	72.1 (101/140)	0.063	87.2 (41/47)	76.5 (39/51)	0.169
Ectopic abortion rate (%)	6.3 (13/207)	3.6 (5/140)	0.264	4.3 (2/47)	2.0 (1/51)	0.510
Chemical pregnancy rate (%)	1.0 (2/207)	5.7 (8/140)	0.009	2.1 (1/47)	3.9 (2/51)	0.607

### 3.3 Logistic regression analysis for miscarriage risk

Logistic regression analysis was performed to analyze the association between PCOS and miscarriage risk (**Table 3**). Univariate analysis indicated that PCOS was significantly associated with an increased risk of miscarriage (OR = 1.795, 95% CI 1.049–3.072, *p* = 0.033). In multivariate analysis, two sequential models were constructed. Model I adjusted for maternal age, BMI, endometrial thickness on hCG day, number of embryos transferred, and embryo transfer method. In this model, PCOS remained an independent risk factor for miscarriage (aOR = 1.802, 95% CI 1.001-3.425, *p* = 0.050). Model II included additional adjustment for the fertilization method (conventional IVF vs. ICSI). In this fully adjusted model, PCOS persisted as a significant predictor (aOR = 1.851, 95% CI 1.019–3.362, *p* = 0.043). Among the covariates, maternal age was also significantly associated with miscarriage in both multivariate model (Model I: aOR = 1.098, 95% CI 1.003–1.201, p = 0.042; Model II: aOR = 1.099, 95% CI 1.005–1.203, p = 0.040), while other variables including maternal BMI, endometrial thickness, number of embryos transferred, embryo transfer method, and fertilization method did not show statistically significant association.

**Table 3:** Logistic regression analysis conducted for miscarriage.

Variable	Univariate analysis		Multivariate analysis (Model I)		Multivariate analysis (Model II)	
	OR (95% CI)	*P*-value	aOR (95% CI)	*P*-value	aOR (95% CI)	*P*-value
PCOS status						
No	Ref		Ref		Ref	
Yes	1.795 (1.049, 3.072)	0.033	1.802 (1.001, 3.425)	0.050	1.851 (1.019, 3.362)	0.043
Maternal age	1.088 (1.000, 1.184)	0.050	1.098 (1.003, 1.201)	0.042	1.099 (1.005, 1.203)	0.040
Maternal BMI	1.077 (1.000, 1.160)	0.050	1.042 (0.962, 1.129)	0.315	1.044 (0.963, 1.131)	0.298
Endometrial thickness (mm) on hCG day	1.001 (0.896, 1.117)	0.992	1.009 (0.902, 1.129)	0.880	1.007 (0.900, 1.128)	0.900
No. of embryos transferred	1.087 (0.440, 2.683)	0.857	1.110 (0.442, 2.790)	0.824	1.106 (0.440, 2.779)	0.831
Embryo transfer method						
Fresh Embryo Transfer	Ref		Ref		Ref	
Frozen Embryo Transfer	0.804 (0.410, 1.577)	0.526	0.831 (0.414, 1.668)	0.603	0.833 (0.415, 1.671)	0.606
Fertilization method						
IVF	Ref				Ref	
ICSI	1.071 (0.430, 2.667)	0.882			0.790 (0.304, 2.055)	0.630

The potential confounders considered for the analysis were maternal age, maternal BMI, endometrial thickness (mm) on hCG day, No. of embryos transferred, embryo transfer method and fertilization method. Model I was adjusted for maternal age, maternal BMI, endometrial thickness (mm) on hCG day, No. of embryos transferred and embryo transfer method, whereas Model II was further adjusted for fertilization method.

### 3.4 Subgroup analyses for miscarriage risk

Subgroup analyses were conducted to evaluate the correlation between PCOS and miscarriage across key demographic and clinical variables, including maternal age, BMI, and embryo transfer method (**Table 4**). None of the subgroups demonstrated a statistically significant association between PCOS and miscarriage. However, several subgroups exhibited elevated odds ratios that approached, but did not meet statistical significance. These included women aged 20–34 years compared to those aged 35–40 years (OR = 1.720, 95% CI 0.996–2.971, *p* = 0.052) and overweight women (BMI ≥ 25 and < 30 kg/m^2^) compared to those with normal BMI (BMI ≥ 18 and < 25 kg/m^2^) (OR = 3.321, 95% CI 0.996–11.074, *p* = 0.051).

**Table 4:** Subgroup analyses of the correlation between PCOS and miscarriage.

Variable	Label	Control		PCOS		OR (95%CI)	*P*-value
		Miscarriage	Total	Miscarriage	Total		
Age (year)	20-34	27	238	43	187	1.720 (0.996, 2.971)	0.052
	35-40	1	16	2	4	5.000 (0.240, 104.147)	0.299
BMI(kg/m^2^)	Normal (≥18.5, <25)	22	196	25	116	1.461 (0.742, 2.875)	0.273
	Overweight (≥25, <30)	3	30	17	52	3.321 (0.996, 11.074)	0.051
Embryo transfer method	Fresh embryo transfer	24	207	34	140	1.642 (0.904, 2.983)	0.103
	Frozen embryo transfer	3	47	11	51	3.143 (0.796, 12.410)	0.102

Subgroup analyses were performed by stratifying on each variable separately. No adjustment for multiple comparisons was applied; therefore, the *p*-values are nominal and should be interpreted as exploratory.

### 3.5 Mendelian randomization results

**Figure 2:**
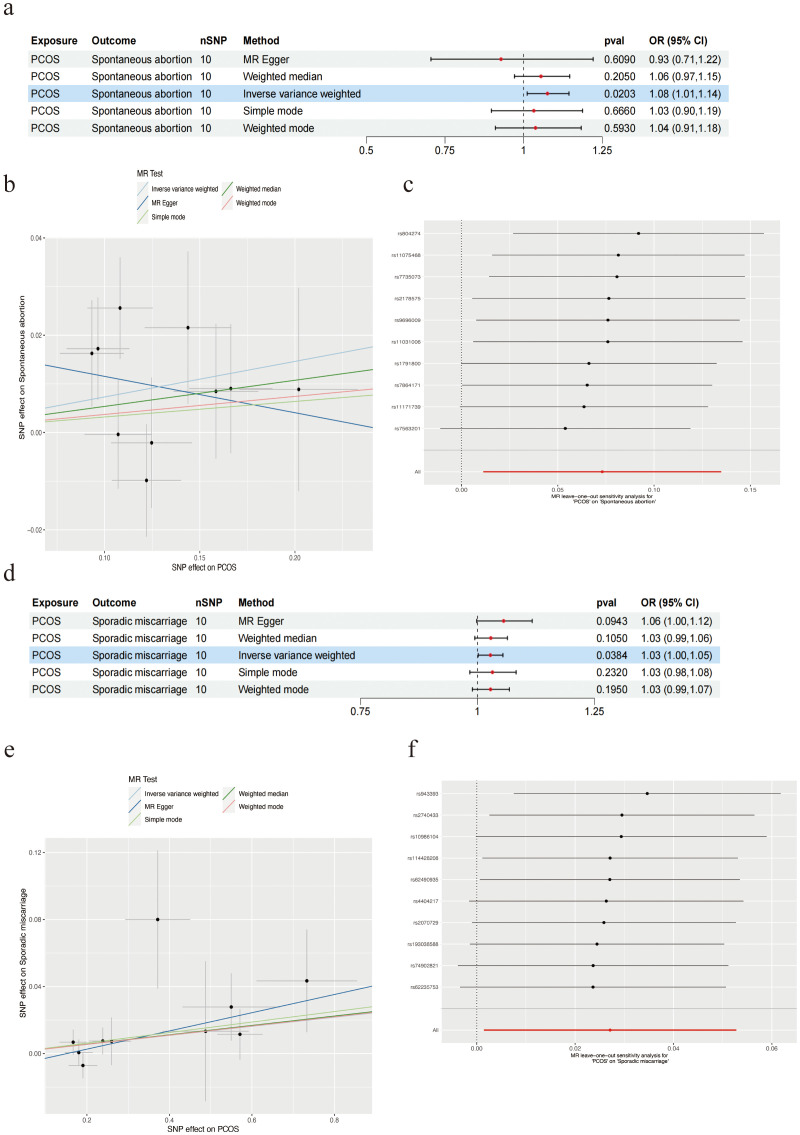
MR analysis of the association between PCOS and miscarriage. **(a-c)** Primary MR analysis of spontaneous abortion. **(a)** Forest plots of five different MR methods. **(b)** Scatter plot of the five methods. **(c)** Leave-one-out sensitivity analysis. **(d-f)** Replication MR analysis of sporadic miscarriage. **(d)** Forest plots of five different MR methods. **(e)** Scatter plot of the five methods. **(f)** Leave-one-out sensitivity analysis.

The primary MR analysis using the IVW method indicated a statistically significant causal relationship between genetically predicted PCOS and an increased risk of spontaneous abortion (OR = 1.08, 95% CI 1.01-1.14, *p* = 0.0203) (**Figure 2a**). This indicates a ~8% higher odds of spontaneous abortion in genetic liability in PCOS women. The positive causal slope estimated by the IVW method was also evident in the scatter plot of SNP effects (**Figure 2b**). Crucially, both the Cochran’s Q test for heterogeneity (Q = 7.92, *p* = 0.44) and the MR-Egger intercept test for directional pleiotropy (intercept = 0.019, *p* = 0.31) were non-significant (**Supplementary Table 4**). These results indicated no substantial heterogeneity among the genetic instruments and no evidence of unbalanced horizontal pleiotropy, supporting the validity of the primary IVW results. The leave-one-out sensitivity analysis further verified that no single SNP drove the association (**Figure 2c**).

To validate our initial findings and enhance robustness, we performed a replication MR analysis in an independent GWAS dataset for sporadic miscarriage using the same set of 10 genetic instruments for PCOS. In the replication MR analysis, a similar causal effect was observed. The IVW method again indicated a significant causal effect of genetically predicted PCOS on sporadic miscarriage (OR = 1.03, 95% CI 1.00 to 1.05, *p* = 0.038), aligning in direction and significance with the primary analysis (**Figure 2d**). Visual inspection of the genetic association scatter plot confirmed this positive causal estimate (**Figure 2e**). Heterogeneity was not observed (Cochran’s Q = 3.73, *p* = 0.67), and the MR-Egger intercept test showed no evidence of directional pleiotropy (intercept = -0.008, *p* = 0.316) (**Supplementary Table 5**). The leave–one-out analysis supported robustness (**Figure 2f**).

## 4 Discussion

To the best of our knowledge, this study represents the first integrated investigation to evaluate the association between PCOS and miscarriage risk by combining a retrospective clinical cohort analysis with MR analyses. Our dual-method strategy yielded convergent evidence supporting a significant and independent causal role of PCOS in increasing the risk of miscarriage.

Clinical data demonstrated that women with PCOS exhibited characteristic endocrine and metabolic profiles, including elevated BMI, prolonged menstrual cycles, abnormal basal hormone levels (increased LH/FSH ratio and hyperandrogenism), and high ovarian reserve. Despite similar numbers of embryos transferred, PCOS patients experienced higher risks of miscarriage and biochemical pregnancy loss, as well as lower live birth rates, with the increased risk predominantly observed in early pregnancy. Univariate regression confirmed PCOS as a miscarriage risk factor, and this association persistent as an independent predictor after adjusting for maternal age, BMI, endometrial thickness on hCG days, number of embryos transferred, embryo transfer method, and fertilization method. Notably, subgroup analyses, though not statistically significant, revealed consistent directional trends. The inclusion of these covariates was deliberate: maternal age reflects oocyte quality decline and embryonic chromosomal abnormalities (22); BMI is associated with metabolic disturbance and insulin resistance, common in PCOS, and linked to increased miscarriage risk (23); Endometrial thickness mirrors implantation receptivity (24). Embryo number, transfer methods, and fertilization method may affect miscarriage risk through embryo quality and implantation dynamics (25). Adjusting for these variables reduces potential confounding and allows for a more accurate estimation of the independent effect of PCOS. Beyond observational associations, our MR analyses provided genetic evidence of causality. Single-source MR can be influenced by weak instrument bias or population-specific effects (26), replication across independent datasets mitigates biases inherent to single-source MR, strengthening confidence in the causal inference. Furthermore, in the replication Mendelian randomization analysis, a relaxed threshold (p < 5×10^-6^) was used to select instrumental variables due to the limited number of genome-wide significant SNPs for PCOS. Although all SNPs had F-statistics >10, the use of a lower threshold may introduce potential weak instrument bias and attenuate causal estimates. However, consistent results across sensitivity analyses and replication datasets support the robustness of our findings. These results should therefore be interpreted with caution, and larger GWAS studies are needed to identify stronger genetic instruments.

Previous observational studies have consistently reported an increased risk of miscarriage among women with PCOS, even after adjusting for age and BMI (3, 7). While these studies provide robust evidence of an association, they remain limited by potential residual confounding from unmeasured biological, endocrine, or embryonic factors and cannot establish causality. In contrast, our study extends these findings by combining a clinical cohort with MR analyses, using genetic variants as instrumental variables to infer the causal effect of PCOS on miscarriage risk. In addition, our finding that PCOS primarily elevates early miscarriage risk (with no late miscarriage effect) aligns with reports that PCOS-related losses cluster in early gestation (5, 27). Regarding embryo transfer strategy, conflicting literature exists: some advocate FET for PCOS to improve outcomes (28), whereas others find no miscarriage difference between fresh ET and FET (29). Our data support the latter, indicating transfer protocol effects may depend on patient characteristics (e.g., endometrial preparation, hormonal milieu), underscoring the need for personalized approaches.

A previous MR study reported no statistically significant association between genetically predicted PCOS and miscarriage risk (30). This heterogeneity may be attributed to differences in GWAS sample size, variability in genetic instruments, definitions of miscarriage, population characteristics, and potential residual pleiotropy. Nevertheless, the direction and magnitude of the causal estimates were broadly consistent, suggesting that differences are likely driven by statistical power and genetic instrument construction rather than conflicting causal inference. Furthermore, our findings, supported by replication and sensitivity analyses, provide further evidence for a potential causal association between PCOS and miscarriage.

The biological mechanisms underlying increased miscarriage risk in PCOS likely stems from multifactorial pathophysiology. Hyperandrogenism disrupts oocyte quality, disrupt early embryonic development, and interfere with endometrial decidualization (31). Insulin resistance exacerbates androgen excess and impairs embryonic energy metabolism and placental vascular development (32). Additional contributors include impaired endometrial receptivity, chronic low-grade inflammation, dyslipidemia, and oxidative stress—all of which compromise embryo–endometrium crosstalk and early placental development (33–35). Collectively, these factors provide a plausible pathophysiological basis for elevated miscarriage risk in PCOS and suggest that clinical management should target multiple mechanisms.

Establishing PCOS as an independent causal risk factor for miscarriage has critical clinical implications. First, it mandates proactive preconception counseling for PCOS patients, emphasizing lifestyle modification (e.g., weight management, insulin sensitizers) and early pregnancy surveillance. Second, clinicians should consider PCOS not merely as a metabolic or infertility diagnosis, but also as a significant predictor of adverse pregnancy outcomes, warranting multidisciplinary care (endocrinology, reproductive medicine). Third, interventions aimed at modifying core features of PCOS—such as anti-androgens, metformin, or ovulation induction protocols optimizing oocyte quality—may prove more effective than generic miscarriage prevention strategies.

Regarding clinical management, metformin has been widely used in PCOS management and may improve insulin sensitivity and ovulatory function. Previous study suggested that metformin did not reduce the risk of miscarriage in women with PCOS (36); however, more recent evidence increasingly supports a potential protective effect, indicating that metformin treatment during pregnancy may be associated with a reduced miscarriage rate in PCOS (37–41). In addition, lifestyle interventions, including weight management, dietary modification, and increased physical activity, are considered first-line strategies for improving insulin resistance and hyperandrogenism in PCOS, which may in turn contribute to improved reproductive outcomes (42). Evidence from randomized controlled trials suggests that such interventions can improve metabolic profiles, ovulation, and pregnancy rates (43). However, current evidence regarding their effect on miscarriage risk remains limited and inconsistent, and whether these benefits extend to miscarriage prevention requires further investigation. Our retrospective design cannot directly assess intervention effects, but our findings provide a rationale for future prospective studies.

Although this retrospective study was conducted in an IVF population, caution is warranted when extrapolating these findings to women with PCOS who conceive naturally. IVF and non-IVF populations differ in several important aspects, including hormonal environment, embryo selection, and the use of adjunctive treatments. These differences may influence miscarriage risk and limit direct generalizability. Nevertheless, previous population-based studies of naturally conceived pregnancies have reported higher miscarriage rates in women with PCOS, suggesting that the increased risk observed in our study may partly reflect the intrinsic pathophysiology of PCOS rather than ART-related factors alone (38). Therefore, further studies in spontaneous conception cohorts are warranted to validate the external applicability of our findings.

Several limitations merit acknowledgement. First, the retrospective cohort design introduces potential residual confounding (e.g., unmeasured lifestyle factors, unadjusted insulin resistance and androgen levels) and selection bias, despite multivariate adjustment. In addition, the control group consisted of infertile women with tubal factor who may have tubal pathology that could influence endometrial receptivity and pregnancy outcomes, potentially introducing bias. Second, MR analyses relied on European-ancestry GWAS data, limiting generalizability to non-European populations such as Asian populations, especially as the study cohort consisted of Chinese participants, future studies in diverse cohorts are needed. Third, PCOS phenotypes were not further stratified in the present study. Given that different phenotypes may be associated with varying miscarriage risks, this aspect warrants investigation in future research.

## 5 Conclusions

In conclusion, by integrating clinical and genetic evidence, our study demonstrates that PCOS is associated with increased miscarriage risk, particularly in early pregnancy, and likely exerts an independent effect beyond conventional risk factors. These findings highlight the need for targeted preconception and early pregnancy management strategies in PCOS patients and provide a basis for future mechanistic and interventional studies.

## Data Availability

The raw data supporting the conclusions of this article will be made available by the authors, without undue reservation.
