# Supplementary material for: Polycystic ovary syndrome and miscarriage risk: dual evidence from cohort study and Mendelian randomization
Source: Front Endocrinol (Lausanne). 2026 Jul 24;17:1854797. doi: 10.3389/fendo.2026.1854797 (PMC13447199; doi:10.3389/fendo.2026.1854797)
Supplement: Supplementary file 1 [file Supplementaryfile1.pdf]

Supplemental Materials

Table 1 The GWAS sources of exposure and outcomes.

MR Analysis	Phenotype	No of. Cases	No. of Controls	GWAS ID/PMID	Consortium /Cohort	Population
Primary MR Analysis	PCOS	10,074	103,164	30566500	7 cohorts of European descent	European
	Spontaneous abortion	20,775	180,063	finngen_R11_O15_ABORT_SPONTAN	FinnGen	
Replication MR Analysis	PCOS	1,909	241,998	finngen_R11_E4_PCOS	FinnGen	
	Sporadic miscarriage	49,996	174,109	GCST011888	European descent	

Table 2 The detailed information of identified SNPs in exposure and outcomes in the primary MR analysis.

SNP					Exposure (PCOS)				Outcome (Spontaneous abortion)				
RS ID	Chr.	Position	EA	OA	Beta	SE	*p*-Value	EAF	Beta	SE	*p*-Value	EAF	F
rs11031006	11	30204981	G	A	0.1585	0.0223	1.06E-12	0.1467	0.00846326	0.0138808	0.542053	0.168297	50.51831
rs11075468	16	52344261	C	T	0.1247	0.0213	5.00E-09	0.1758	-0.00211599	0.0134154	0.87467	0.186006	34.2747
rs11171739	12	56076841	C	T	-0.0965	0.0165	5.39E-09	0.5803	-0.0172355	0.0105359	0.101864	0.569547	34.20478
rs1791800	11	114079018	G	A	-0.1438	0.0228	2.67E-10	0.1753	-0.0215537	0.0156752	0.169126	0.127688	39.77847
rs2178575	2	212527042	G	A	0.1663	0.0219	3.34E-14	0.1512	0.00903068	0.0132858	0.496679	0.190589	57.66287
rs7563201	2	43334641	G	A	-0.1081	0.0172	3.68E-10	0.4507	-0.0255907	0.0104625	0.0144474	0.476584	39.49976
rs7735073	5	132479665	G	A	-0.1071	0.0177	1.47E-09	0.3284	0.000387996	0.0111564	0.972257	0.32919	36.61275
rs7864171	9	94960984	G	A	-0.0933	0.0168	2.95E-08	0.4284	-0.016262	0.0109152	0.136263	0.353064	30.84216
rs804274	8	11767696	C	A	-0.122	0.0182	2.07E-11	0.7095	0.0098441	0.0115773	0.395162	0.717862	44.93419
rs9696009	9	123856954	G	A	0.202	0.0311	7.96E-11	0.0679	0.00886065	0.0209037	0.671653	0.0660396	42.18732

Chr. : Chromosome； EA: effect allele; OA: other allele; EAF: effect allele frequency

Table 3 The detailed information of identified SNPs in exposure and outcomes in the replication MR analysis.

SNP					Exposure (PCOS)				Outcome (Sporadic miscarriage)				
RS ID	Chr.	Position	EA	OA	Beta	SE	*p*-Value	EAF	Beta	SE	*p*-Value	EAF	F
rs10986104	9	123786056	T	C	0.570993	0.0550463	3.29E-25	0.0661143	0.0115	0.0151	0.4479	0.0625	107.59827
rs114428208	2	29852984	T	C	0.487741	0.104639	3.14E-06	0.0185256	0.0133	0.0418	0.7503	0.0089	21.72658
rs193038588	4	28256871	T	C	-0.371617	0.0787637	2.38E-06	0.0549877	-0.08	0.0412	0.052	0.0588	22.26071
rs2070729	5	132484229	A	C	-0.166717	0.0327441	3.55E-07	0.502836	-0.0068	0.0075	0.3645	0.4434	25.9235
rs2740433	8	11763941	A	G	-0.180359	0.0340083	1.14E-07	0.674246	-6.00E-04	0.0078	0.9366	0.6573	28.12586
rs4404217	2	212521093	A	G	0.238488	0.0334812	1.06E-12	0.342483	0.0075	0.008	0.3458	0.3073	50.73773
rs62235753	22	28876649	T	C	0.733097	0.121985	1.86E-09	0.011916	0.0434	0.0307	0.1571	0.0169	36.11686
rs62490935	8	11594798	T	C	0.259861	0.0471103	3.47E-08	0.121448	0.0073	0.0142	0.6052	0.0751	30.42639
rs74902821	1	192763058	A	G	0.549877	0.118421	3.43E-06	0.0130772	0.0278	0.0201	0.168	0.0396	21.56124
rs943393	9	94737144	T	C	0.190408	0.0348491	4.66E-08	0.652943	-0.0071	0.0077	0.3574	0.5902	29.85295

Chr. : Chromosome； EA: effect allele; OA: other allele; EAF: effect allele frequency

Table 4 Causal association between PCOS and Spontaneous abortion in the primary MR analysis: heterogeneity and pleiotropy

Outcomes	No. of IVs	Heterogeneity test		MR-Egger pleiotropy test		
		Q	*p*-Value	Intercept	SE	*p*-Value
Spontaneous abortion	10	7.921	0.441	0.0190	0.018	0.311

Table 5 Causal association between PCOS and Spontaneous abortion in the replication MR analysis: heterogeneity and pleiotropy

Outcomes	No. of IVs	Heterogeneity test		MR-Egger pleiotropy test		
		Q	*p*-Value	Intercept	SE	*p*-Value
Sporadic miscarriage	10	5.754	0.675	-0.008	0.008	0.316
